# Highly Pathogenic Avian Influenza A(H5N1) Virus–Induced Mass Death of Wild Birds, Caspian Sea, Russia, 2022

**DOI:** 10.3201/eid2912.230330

**Published:** 2023-12

**Authors:** Ivan Sobolev, Alimurad Gadzhiev, Kirill Sharshov, Olesia Ohlopkova, Kristina Stolbunova, Artem Fadeev, Nikita Dubovitskiy, Alexandra Glushchenko, Victor Irza, Maxim Perkovsky, Kirill Litvinov, Natalia Meshcheriakova, Guy Petherbridge, Alexander Shestopalov

**Affiliations:** Federal Research Center of Fundamental and Translational Medicine, Novosibirsk, Russia (I. Sobolev, K. Sharshov, O. Ohlopkova, K. Stolbunova, N. Dubovitskiy, A. Glushchenko, A. Shestopalov);; Dagestan State University, Makhachkala, Russia (A. Gadzhiev);; Smorodintsev Research Institute of Influenza, Saint Petersburg, Russia (A. Fadeev);; Federal Governmental State-Financed Institution Federal Centre for Animal Health, Vladimir, Russia (V. Irza);; Astrakhan State Biosphere Nature Reserve, Astrakhan, Russia (M. Perkovsky, K. Litvinov, N. Meshcheriakova);; Caspian Center for Nature Conservation, Makhachkala (G. Petherbridge)

**Keywords:** highly pathogenic avian influenza, H5N1, HPAI virus, waterbirds, wild birds, outbreak, clade 2.3.4.4.b, mass death, influenza, respiratory infections, viruses, zoonoses, Maliy Zhemchuzhniy Island, Caspian Sea, Russia

## Abstract

In May 2022, we observed a substantial die-off of wild migratory waterbirds on Maliy Zhemchuzhniy Island in the Caspian Sea, Russia. The deaths were caused by highly pathogenic avian influenza A(H5N1) clade 2.3.4.4.b virus. Continued surveillance of influenza viruses in wild bird populations is needed to predict virus spread over long distances.

The coastal and estuarine wetlands of the northern Caspian Sea, which borders southeast Russia, provide support for millions of waterfowl and shorebirds during nesting, molting, migration, and wintering periods (*1*,*2*); >300 species of birds are found in this region. The area is crossed by several migration flyways (Figure 1), of which the Black Sea/Mediterranean Flyway is the main migratory route (*3*). The region plays a critical role in the reproduction of colonial nesting birds, such as pelicans, cormorants, herons, gulls, and terns.

**Figure 1:**
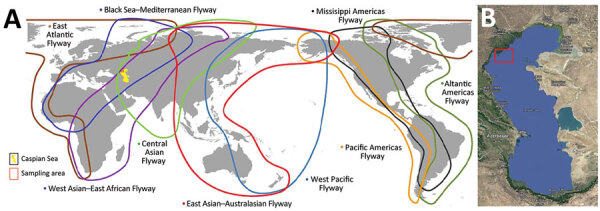
Major bird migration flyways (A) and sampling area of birds (B) in study of highly pathogenic avian influenza A(H5N1) virus–induced mass death of wild birds, Caspian Sea, Russia, 2022. Map of migration routes was provided online by the East Asian–Australasian Flyway Partnership (https://www.eaaflyway.net/the-flyway). Yellow shading in panel A indicates the location of the Caspian Sea; red rectangles in both panels indicate sampling location of dead birds on Maliy Zhemchuzhniy Island.

Maliy Zhemchuzhniy Island is located in the northern part of the Caspian Sea (Figure 1). Monitoring data on waterbirds has shown the high ecologic importance of this area, not only during the nesting period but also during bird migration. The island has had >150 species of birds registered since 2016. A breeding colony of Caspian gulls is located on the island, along with colonies of Great black-headed gulls and Caspian terns, which are all listed in the Red Data Book of Russia. We investigated mass deaths of wild migratory waterbirds on Maliy Zhemchuzhniy Island that occurred in May 2022. The study was approved by the Committee on Biomedical Ethics at the Federal Research Center of Fundamental and Translational Medicine in Novosibirsk, Russia (protocol nos. 2013-23, 2019-3, and 2021-10).

## The Study

On April 28, 2022, near the end of the egg incubation period, we had counted a total of 26,769 Great black-headed gull nests, 7,340 Caspian gull nests, and 5,267 Caspian tern nests on Maliy Zhemchuzhniy Island. In May, 1 week later, we detected mass deaths of waterbirds on the island comprising 25,157 Great black-headed gulls, 3,507 Caspian gulls, 5,641 Caspian terns, and 14 Dalmatian pelicans (Appendix 1 Figure 1). Nearly all gull and tern chicks died during the nesting period. The mass death event began during hatching of Great black-headed gulls. We only found the corpses of chicks (with down but without feathers) that were similar in age. We assume that not all of the chicks actually died from disease; death of adult birds likely led to the deaths of chicks in their nests. The Caspian terns were still incubating eggs at that time; consequently, the death of adult terns led to the death of egg clutches in their nests. We did not observe live chicks on the island during the remaining 2022 nesting season.

**Figure 2:**
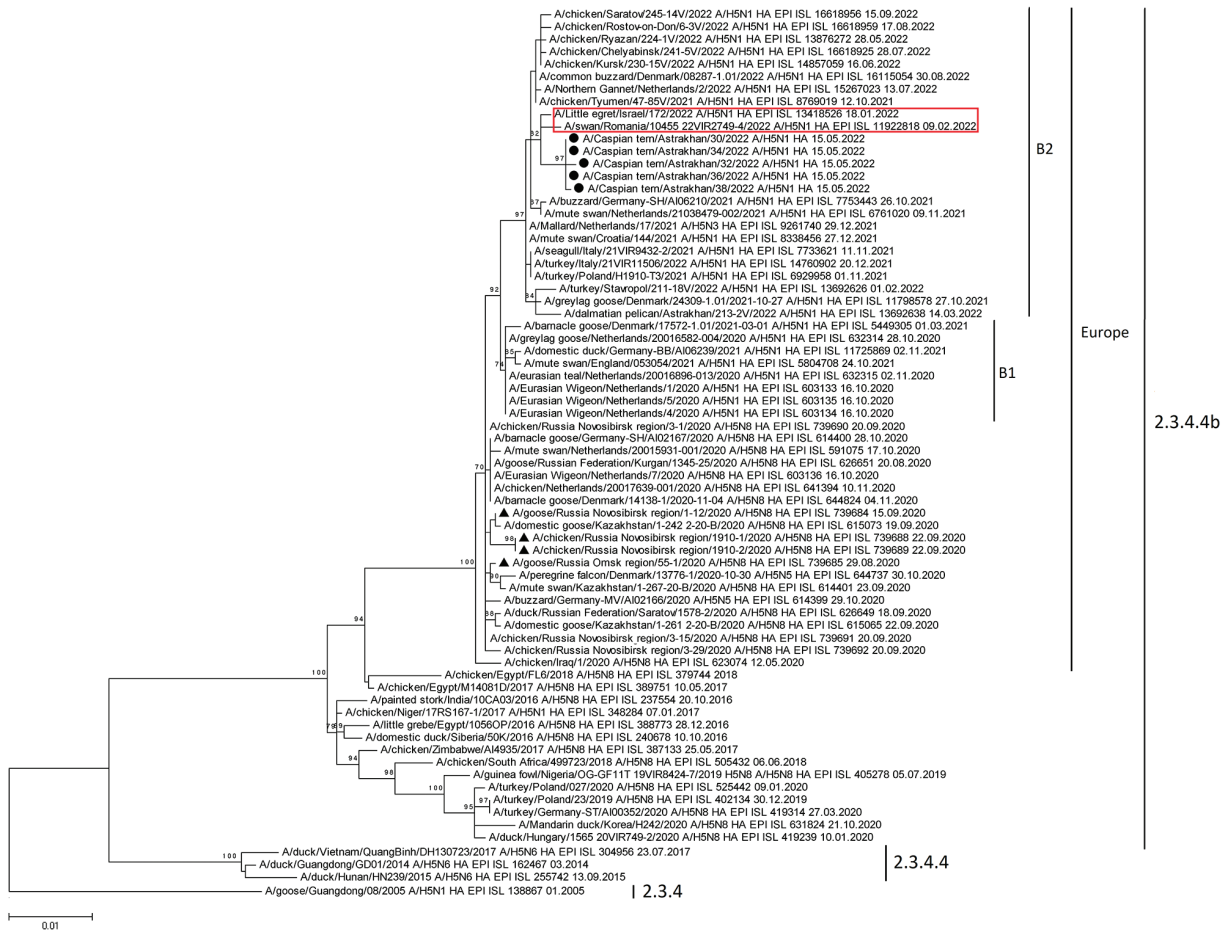
Phylogenetic analysis of viruses isolated from 5 dead Caspian terns in study of highly pathogenic avian influenza A(H5N1) virus–induced mass death of wild birds, Caspian Sea, Russia, 2022. Maximum-likelihood phylogenetic tree was constructed for hemagglutinin gene segments. Black circles indicate highly pathogenic avian influenza (HPAI) A H5N1 virus strains isolated from the Caspian Sea region; black triangles indicate Egyptian-like HPAI virus strains from Russia isolated in 2020; red box indicates HPAI strains from Israel and Romania that were closely related to viruses from the Caspian Sea. Viruses belonging to clade 2.3.4.4b and B1 or B2 sublineages and those with hemagglutinin genes found in Europe are indicated. Sequences were obtained from the GISAID EpiFlu database (https://www.gisaid.org); identification numbers are provided. Scale bar indicates nucleotide substitutions per site.

In May 2022, we collected 10 samples from deceased Caspian terns on the island. All samples tested positive for H5Nx avian influenza virus (AIV) by real-time PCR. We characterized 5 isolates by using complete genome sequencing, phylogenetic analysis, and intravenous pathogenicity index testing (Table). We identified all 5 isolates as highly pathogenic avian influenza (HPAI) viruses, according to the amino acid sequence of the hemagglutinin (HA) polybasic proteolytic cleavage site (PLREKRRKR/G) (Appendix 1 Figure 2) and according to intravenous pathogenicity index values of 2.92–2.93 (reference values were for chickens) (Appendix). We determined the HPAI neuraminidase (NA) subtype was N1 by using sequence analysis. 

**Table:** Sequenced viruses isolated from Caspian terns on Maliy Zhemchuzhniy Island in study of highly pathogenic avian influenza A(H5N1) virus–induced mass death of wild birds, Caspian Sea, Russia, 2022*

Isolate	Location	Sample type	Collection date	IVPI	GISAID no.
A/Caspian_tern/Astrakhan/30/2022	45°02′ N, 48°19′ E	Intestine	2022 May 15	2.92	EPI_ISL_16020401
A/Caspian_tern/Astrakhan/32/2022	45°02′ N, 48°19′ E	Intestine	2022 May 15	2.92	EPI_ISL_16020402
A/Caspian_tern/Astrakhan/34/2022	45°02′ N, 48°19′ E	Intestine	2022 May 15	2.93	EPI_ISL_16020403
A/Caspian_tern/Astrakhan/36/2022	45°02′ N, 48°19′ E	Liver	2022 May 15	2.93	EPI_ISL_16020404
A/Caspian_tern/Astrakhan/38/2022	45°02′ N, 48°19′ E	Liver	2022 May 15	2.92	EPI_ISL_16020405

*Nucleotide sequences were deposited in the GISAID database (https://www.gisaid.org). IVPI, intravenous pathogenicity index.

The A/goose/Guangdong/1/96 (Gs/GD) strain, which was isolated in 1996 from a domestic goose, is considered the ancestor of AIV carrying the highly pathogenic H5 HA subtype (*4*,*5*). The Gs/GD lineage of HPAI H5N1 viruses evolved into several sublineages that subsequently reassorted with low pathogenicity avian influenza (LPAI) viruses, leading to the formation of H5Nx reassortant variants (*6*,*7*). Viruses of the Gs/GD lineage initially circulated in southern China. However, during 2004–2005, they began spreading throughout Asia, Europe, the Middle East, and Africa (*8*). An increased number of available sequences led to the creation of the H5 virus classification and identification of 10 clades (*4*,*8*–*13*). Subsequently, clade 2 was split into 5 subclades, each containing numerous genetic subgroups. Clade 2.3.4.4 is dominant and has been divided into several genetic subgroups, including 2.3.4.4.b, which, in turn, diverged into 2 sublineage branches, B1 and B2 (*14*). According to phylogenetic analysis of HA segments, we found the Caspian Sea strains (the AIV strains isolated from dead birds in the Caspian Sea region) belonged to HPAI H5N1 clade 2.3.4.4.b (Figure 2). Furthermore, we found the HPAI H5N1 virus isolates from Maliy Zhemchuzhniy Island belonged to the B2 sublineage because isoleucine was present at position 548 in HA (*14*).

The polymerase basic (PB) 1, polymerase acidic (PA), HA, nucleoprotein (NP), NA, and matrix (M) protein gene segments of the Caspian Sea strains were phylogenetically related to H5N1 viruses previously identified in wild birds in Israel in January 2022. However, all 8 gene segments were similar to those of strains from Romania isolated in February 2022 (Appendix 1 Figures 3–9). The phylogenetic relationships of PB2 and nonstructural (NS) gene segments between AIV strains from Israel and Romania remains unclear, because no sequences were available for the segments from Israel in the GISAID EpiFlu database (https://www.gisaid.org).

## Conclusions

On the basis of virus phylogeny and chronology of virus detection, we hypothesize that the HPAI virus found in the Caspian Sea region was present in birds migrating to their wintering sites during autumn 2021 and was detected in Israel during the winter months of 2022. During spring migration in 2022, the virus strain spread from the Middle East to nesting areas, leading to wild bird deaths on Maliy Zhemchuzhniy Island. Because of the lack of some genetic data on closely related viruses, it remains unclear whether the Caspian Sea strains were transmitted through Europe (Romania) from Israel or directly from Israel. 

The HPAI H5N1 viruses detected during the mass death of birds on Maliy Zhemchuzhniy Island evolved from sequential reassortment of multiple genetic variants of LPAI and HPAI viruses (Appendix 1 Figures 3–9). The new variants probably acquired M and HA gene segments from viruses (Egyptian-like) detected in Siberia and Kazakhstan in 2020 (*15*). PB2, PB1, PA, NP, and NA gene segments from HPAI viruses likely emerged as a result of reassortment with LPAI viruses during 2020–2021; NS segments likely emerged from LPAI viruses detected during 2021–2022. NS sequences closely related to those of strains isolated in the Caspian Sea regions and Romania were found in LPAI viruses predominantly circulating in Asia during 2019–2021 (Appendix 1 Figure 9). HPAI viruses with such NS sequences have been identified only in Romania and the Caspian Sea.

Gene segments of HPAI H5N1 viruses from the Caspian Sea were closely related to virus segments found in different parts of Eurasia. Specifically, PB1, PA, HA, NA, and M protein gene segments were predominantly related to those in Europe, whereas related NP and NS segments were more prevalent in Asia. In addition, the PA segment from the Caspian Sea strains was also identified in Africa, and PB2 was related to PB2 of viruses detected in the Far East (Japan, Korea, and China), Siberia (Novosibirsk region), Bangladesh, and Europe (Italy, Slovenia, and Czech Republic). Three major flyways pass through the Caspian Sea region: the Black Sea/Mediterranean Flyway, the West Asian–East African Flyway, and the Central Asian Flyway. However, we found that gene segments of HPAI viruses from the Caspian Sea were related to variants identified in the Far East, indicating widespread distribution and exchange of influenza virus genes well beyond the major flyways. Therefore, continued surveillance and monitoring of AIVs (primarily HPAI viruses) in wild bird populations will be needed worldwide to track and predict the spread of these viruses over long distances.

Appendix 1 Additional information for highly pathogenic avian influenza A(H5N1) virus–induced mass death of wild birds, Caspian Sea, Russia, 2022.

Appendix 2 Acknowledgment for GISAID EpiFlu sequences used for highly pathogenic avian influenza A(H5N1) virus–induced mass death of wild birds, Caspian Sea, Russia, 2022.
