# Supplementary material for: Highly Pathogenic Avian Influenza A(H5N1) Virus–Induced Mass Death of Wild Birds, Caspian Sea, Russia, 2022
Source: Emerg Infect Dis. 2023 Dec;29(12):2528–32. doi: 10.3201/eid2912.230330 (PMC10683809; doi:10.3201/eid2912.230330)
Supplement: Appendix 1 — Additional information for highly pathogenic avian influenza A(H5N1) virus–induced mass death of wild birds, Caspian Sea, Russia, 2022. [file 23-0330-Techapp-s1.pdf]

Article DOI: https://doi.org/10.3201/eid2912.230330

EID cannot ensure accessibility for supplementary materials supplied by authors. Readers who have difficulty accessing supplementary content should contact the authors for assistance.

Highly Pathogenic Avian Influenza A(H5N1) Virus–Induced Mass Death of Wild Birds, Caspian Sea, Russia, 2022

Appendix 1

Additional Materials and Methods

Sampling Area

The Caspian Sea region is a crucial area for avian influenza virus (AIV) surveillance in Eurasia, because it is situated at the convergence of 3 major bird migratory routes: the Central Asian Flyway, East Africa/West Asia Flyway, and Black Sea/Mediterranean Flyway (Figure 1, main text). Thus, the main migratory pathways of birds from Europe, Africa, and Asia intersect here (*1–3*). Because of the abundance of saltwater bays, lagoons, river deltas, and lake systems, the western coast of the Caspian Sea provides a highly favorable route for the seasonal migrations of numerous bird species in Eurasia (*4*,*5*). The ecologic characteristics of those unique habitats enable wild waterfowl and shorebirds to utilize numerous wetlands in the Caspian Sea region for both wintering and nesting. Moreover, this region also provides potential stopover sites for birds that migrate further into Europe or Africa.

Reconstructive analysis of the flight routes of wild birds in general, and gulls (Laridae) in particular, was performed as previously described (*5*) according to data from the Bird Ringing Center of Russia (https://sev-in.ru/en/bird-ringing-and-scientificinformation-cente) (Appendix 1 Figures 10). As a result, it has been shown that flight routes passing through the Caspian Sea region cover not only the territory of Eurasia but also parts of Africa. They also pass through countries in Europe and the Middle East. Those results enables us to assume that the influenza virus spreads through avian spring migration to the Caspian Sea, either directly from Israel and the Levant or through Europe (likely via Romania, as indicated by phylogenetic analysis). In

addition, analysis of flight routes and migration corridors (notable for their diverse destinations) indicates the significance of the Caspian region in the spread of avian influenza virus and early detection of new variants.

Maliy Zhemchuzhniy Island is located in the northern part of the Caspian Sea near the maritime boundary of the Republic of Kalmykia and the Astrakhan region (latitude 45°02′ N, longitude 48°19′ E). This flat, low-lying island, composed of sand and clam shells, was formed during a regression of the Caspian Sea in the 1930s on the site of an underwater shoal. It is constantly exposed to wind and waves, which cause changes in its configuration, and areas are either completely or partially lacking terrestrial vegetation. During 2016–2022, the size of the island remained relatively constant. In 2021, the island was estimated to be 26.33 ha and had a length of ≈2 km and maximum width of ≈0.3 km.

Samples

In May 2022, we collected 10 samples from Caspian terns on the island during a mass die-off of wild birds. In all of those samples, the matrix protein gene segment of AIV A and the hemagglutinin gene segment of the H5Nx subtype were detected by using multiplex real-time PCR (AmpliSens Influenza virus A H5N1-FRT PCR kit; InterLabService Ltd, https://en.interlabservice.ru). All analyzed viruses were isolated from 10-day-old chicken embryonated eggs by using chicken embryo inoculation. All viruses caused the death of chicken embryos within 2 days. Isolates were shown to be H5 positive by using real-time PCR.

Genome Sequencing and Phylogenetic Analysis

Next-generation sequencing of complete genomes was performed by using the Illumina MiSeq platform (https://www.illumina.com) and associated reagent kits according to the manufacturer's methodology. RNA was extracted by using the QIAamp Viral RNA Mini Kit (https://www.qiagen.com). Whole-genome amplification was performed by using a modified protocol (*6*). DNA libraries were prepared by using a Nextera DNA Flex Library Prep kit (Illumina) and sequenced by using MiSeq Reagent Kit v3 (600-cycle) (Illumina). Consensus sequences were generated by using Bowtie software (https://www.bowtiepro.com).

Nucleotide sequences were deposited in the GISAID database under accession nos. EPI_ISL_16020401–405. Multiple alignments were performed by using MUSCLE (https://www.ebi.ac.uk/Tools/msa/muscle); editing, which included translating the nucleotide

sequences into amino acid sequences, was performed by using BioEdit (https://bioedit.software.informer.com) and UGENE (http://www.ugene.net) software. Initial maximum-likelihood phylogenies for each of the gene segments were generated with RAxML (*7*) by using the general time-reversible nucleotide substitution model. Final dendrograms were generated and visualized by using MEGA5 (*8*). Bootstrap support values were generated by using 1,000 rapid bootstrap replicates.

Intravenous Pathogenicity Index

For determining intravenous pathogenicity index (IVPI) values for 9 viruses, 0.1 mL of a 1:10 dilution of infectious allantoic fluid was intravenously inoculated into ten 6-week-old specific pathogen-free chickens. The IVPI was calculated according to the OIE standard protocol (https://www.woah.org/fileadmin/Home/eng/Health_standards/tahm/3.03.04_AI.pdf). Virus isolates with an IVPI value >1.2 were considered highly pathogenic avian influenza A viruses. The challenge study and all experiments with live viruses were conducted in a Biosafety Level 3 facility.

References

1. Danilenko EA, Soldatov MS. Bird migration in the Caspian region [in Russian], 2015. http://de.geogr.msu.ru/casp

2. Iverson SA, Gavrilov A, Katzner TE, Takekawa JY, Miller TA, Hagemeijer W, et al. Migratory movements of waterfowl in Central Asia and avian influenza emergence: sporadic transmission of H5N1 from east to west. IBIS. 2011;153:279–92. https://doi.org/10.1111/j.1474-919X.2010.01095.x

3. Gulyaeva M, Sobolev I, Sharshov K, Kurskaya O, Alekseev A, Shestopalova L, et al. Characterization of avian-like influenza A(H4N6) virus isolated from Caspian seal in 2012. Virol Sin. 2018;33:449–52. PubMed https://doi.org/10.1007/s12250-018-0053-y

4. Vilkov EV. The genesis and evolution of the Caspian Sea lagoons as avifauna refuges at the transboundary scale. Open J Marine Sci. 2016;6:115–24. https://doi.org/10.4236/ojms.2016.61011

5. Vilkov EV. Changes in the strategy of migration of gulls (Laridae) along the western coast of the Caspian Sea as a result of environmental changes in space and time. Contemp Probl Ecol. 2016;9:233–53. https://doi.org/10.1134/S199542551603015X

6. Zhou B, Donnelly ME, Scholes DT, St George K, Hatta M, Kawaoka Y, et al. Single-reaction genomic amplification accelerates sequencing and vaccine production for classical and swine origin human influenza a viruses. J Virol. 2009;83:10309–13. PubMed https://doi.org/10.1128/JVI.01109-09

7. Stamatakis A. RAxML version 8: a tool for phylogenetic analysis and post-analysis of large phylogenies. Bioinformatics. 2014;30:1312–3. PubMed https://doi.org/10.1093/bioinformatics/btu033

8. Tamura K, Peterson D, Peterson N, Stecher G, Nei M, Kumar S. MEGA5: molecular evolutionary genetics analysis using maximum likelihood, evolutionary distance, and maximum parsimony methods. Mol Biol Evol. 2011;28:2731–9. PubMed https://doi.org/10.1093/molbev/msr121

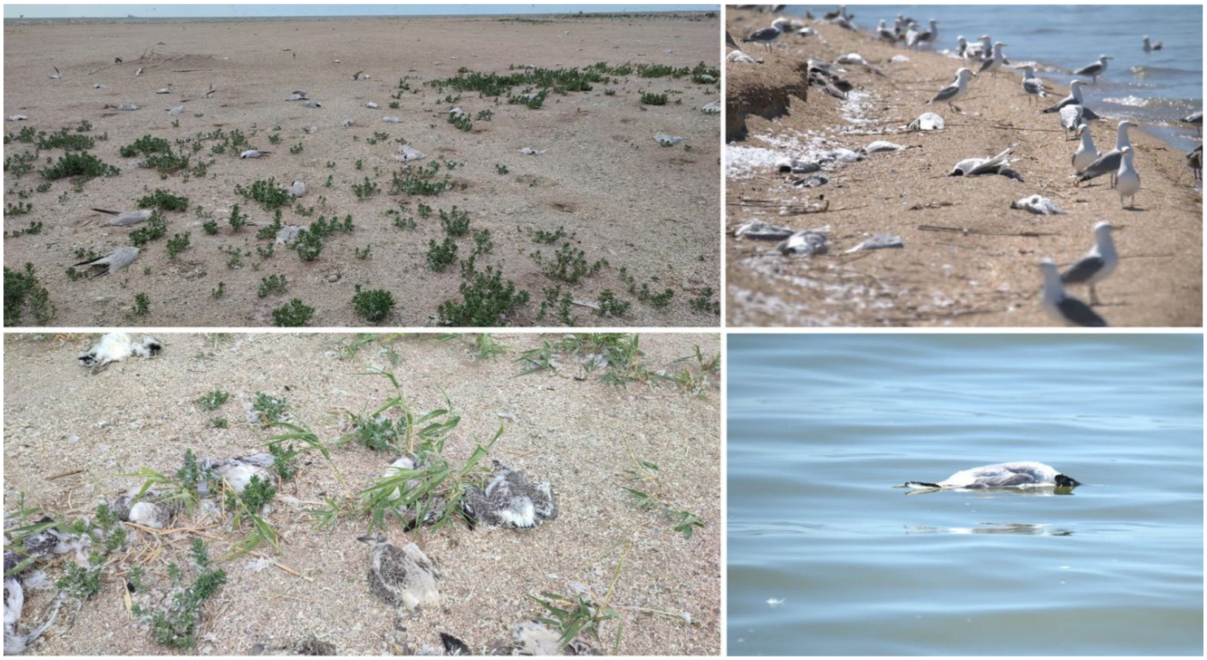

Appendix 1 Figure 1. Dead waterbirds found on Maliy Zhemchuzhniy Island in the northern Caspian Sea, Russia, May 2022.

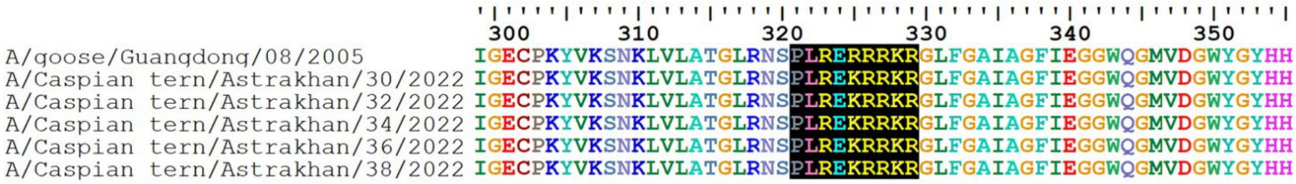

Appendix 1 Figure 2. Amino acid sequences of polybasic cleavage site within hemagglutinin from viruses isolated from dead Caspian terns on Maliy Zhemchuzhniy Island, Caspian Sea, Russia in May 2022. We identified all 5 strains as highly pathogenic avian influenza viruses according to the amino acid sequence of the hemagglutinin proteolytic cleavage site. Black boxes indicate the PLREKRRKR/G cleavage site. Sequences were aligned with an H5N1 highly pathogenic avian influenza virus strain from a goose.

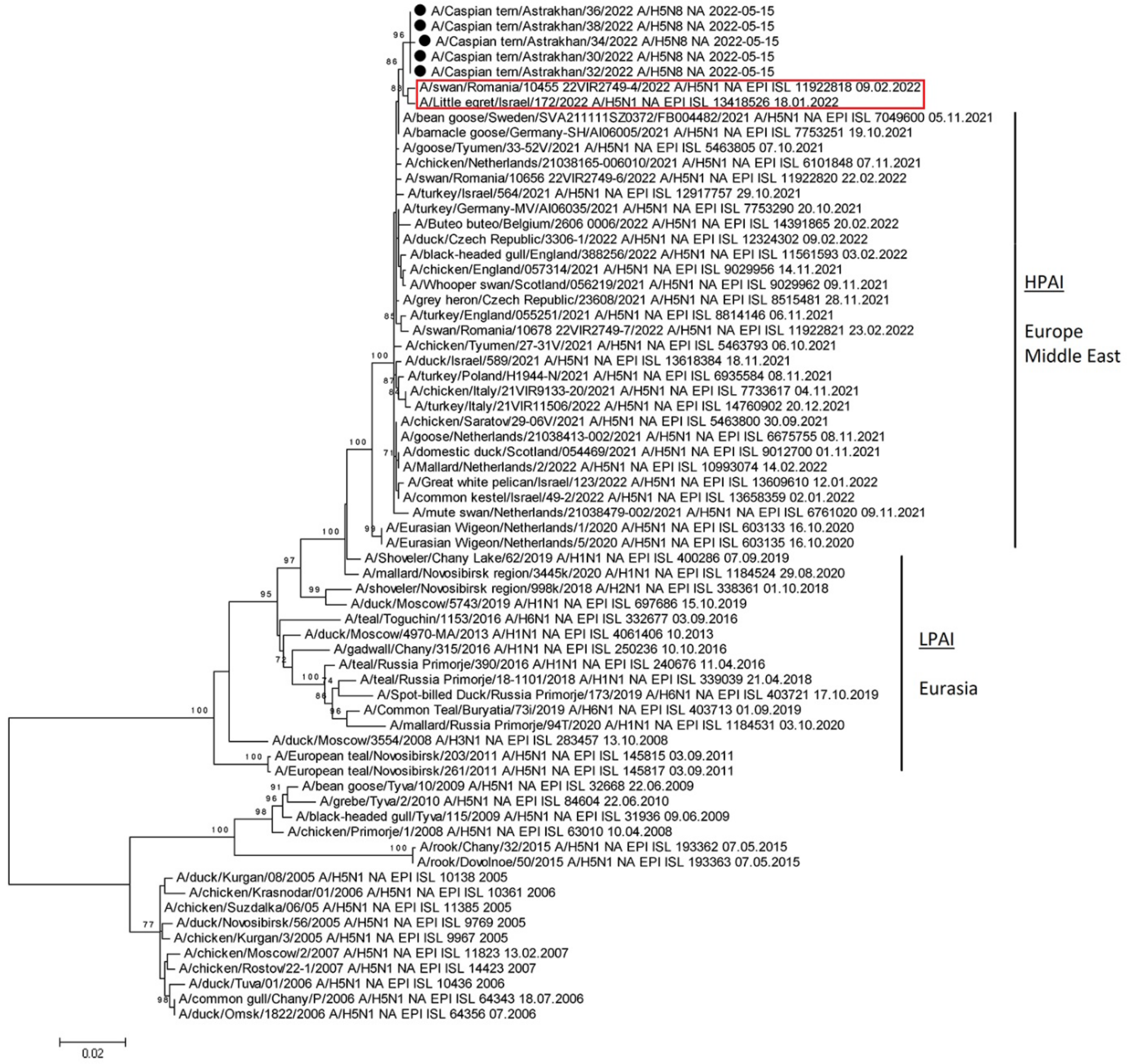

Appendix 1 Figure 3. Phylogenetic analysis of neuraminidase gene segments of avian influenza viruses. Trees were constructed by using the maximum-likelihood method. Closed circles indicate the highly pathogenic avian influenza H5N1 virus strains isolated from dead Caspian terns on Maliy Zhemchuzhniy Island, Caspian Sea, Russia in May 2022. Red box indicates virus strains from Israel and Romania closely related to the Caspian Sea strains. Sequences were obtained from the GISAID EpiFlu database (https://www.gisaid.org). Scale bar indicates nucleotide substitutions per site. HPAI, high pathogenicity avian influenza; LPAI, low pathogenicity avian influenza.

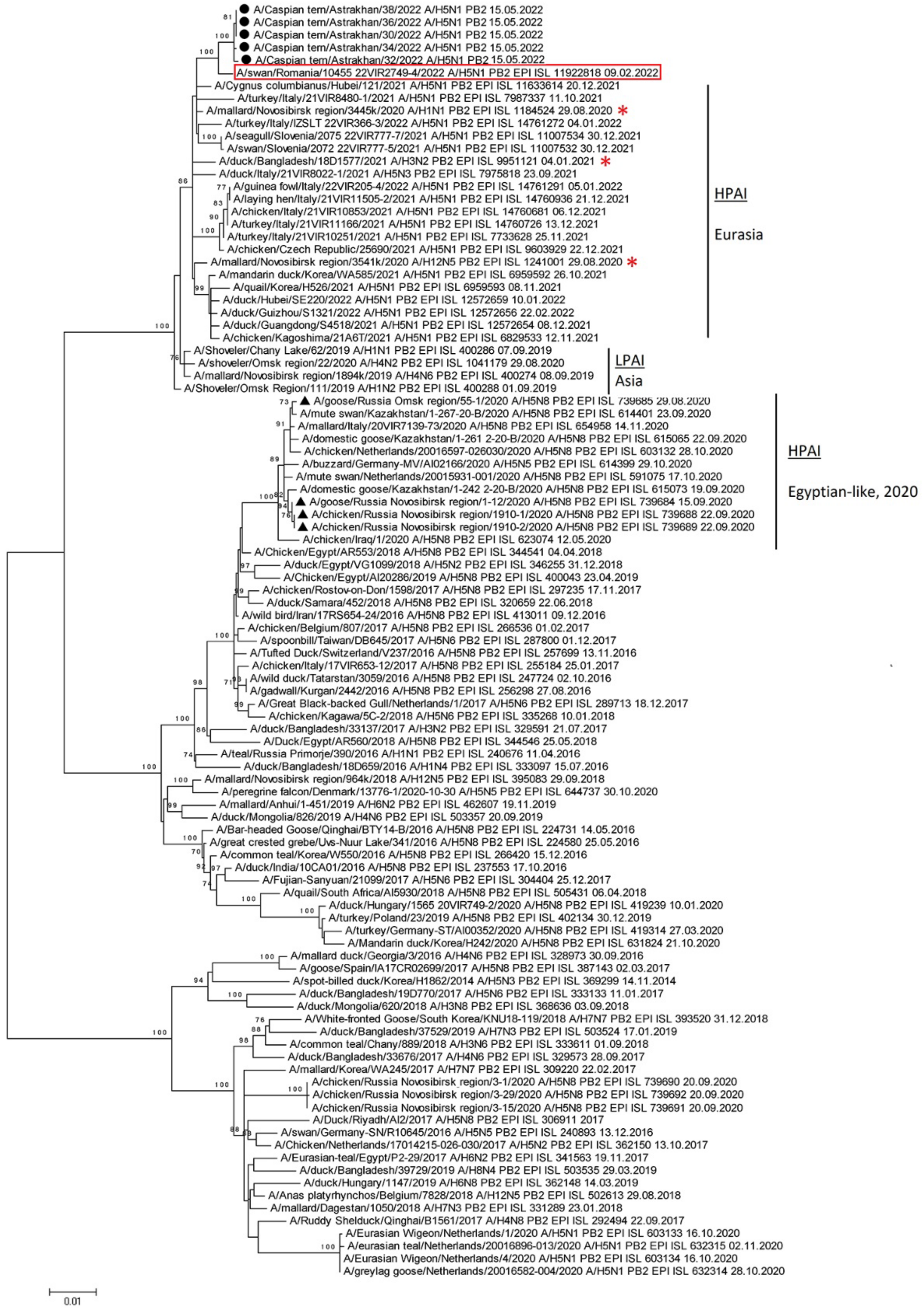

Appendix 1 Figure 4. Phylogenetic analysis of polymerase basic 2 protein gene segments of avian influenza viruses. Trees were constructed by using the maximum-likelihood method. Closed circles indicate the highly pathogenic avian influenza H5N1 virus strains isolated from dead Caspian terns on Maliy Zhemchuzhniy Island, Caspian Sea, Russia in May 2022. Red box indicates virus strains from

Romania closely related to the Caspian Sea strains. Red asterisks indicate low pathogenicity viruses found within highly pathogenic virus groups that were related to the Caspian Sea strains (presumably, highly pathogenic viruses appeared because of reassortment with low pathogenicity viruses). Closed triangles indicate Egyptian-like virus strains from Russia isolated in 2020. Sequences were obtained from the GISAID EpiFlu database (https://www.gisaid.org). Scale bar indicates nucleotide substitutions per site. HPAI, high pathogenicity avian influenza; LPAI, low pathogenicity avian influenza.

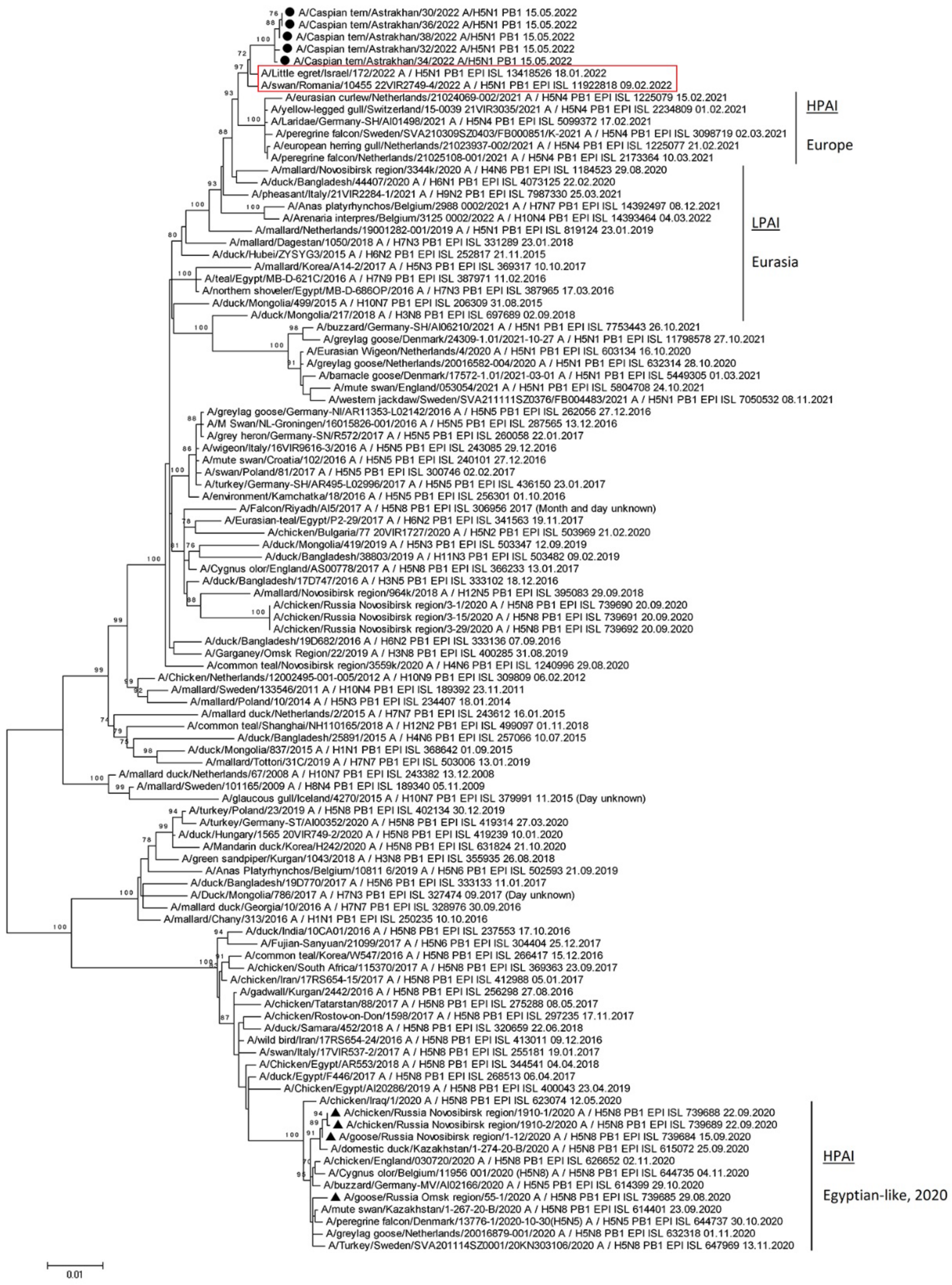

Appendix 1 Figure 5. Phylogenetic analysis of polymerase basic 1 protein gene segments of avian influenza viruses. Trees were constructed by using the maximum-likelihood method. Closed circles indicate the highly pathogenic avian influenza H5N1 virus strains isolated from dead Caspian terns on Maliy Zhemchuzhniy Island, Caspian Sea, Russia in May 2022. Red box indicates virus strains from Israel

and Romania closely related to the Caspian Sea strains. Closed triangles indicate Egyptian-like virus strains from Russia isolated in 2020. Sequences were obtained from the GISAID EpiFlu database (https://www.gisaid.org). Scale bar indicates nucleotide substitutions per site. HPAI, high pathogenicity avian influenza; LPAI, low pathogenicity avian influenza.

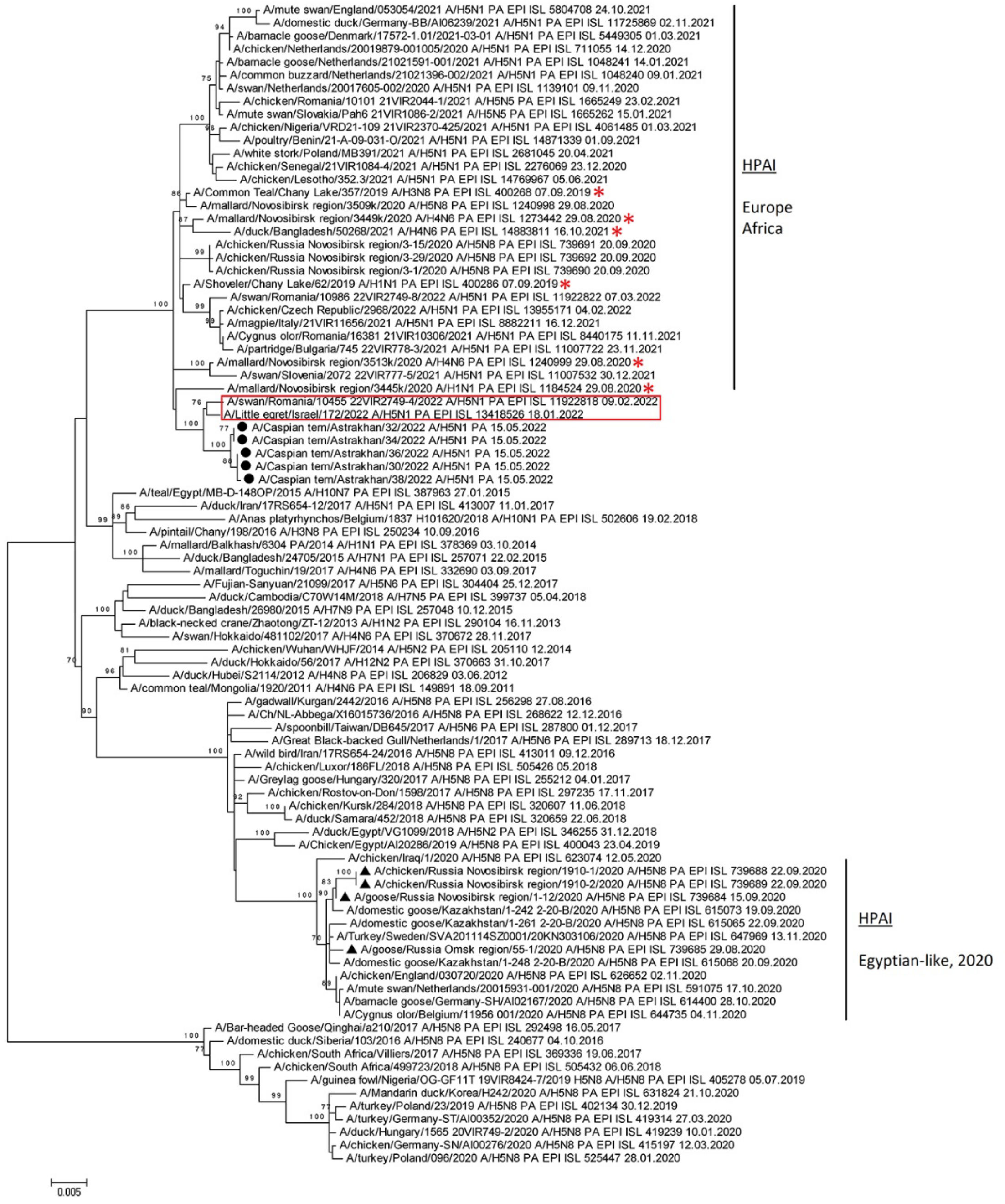

Appendix 1 Figure 6. Phylogenetic analysis of polymerase acidic protein gene segments of avian influenza viruses. Trees were constructed by using the maximum-likelihood method. Red asterisks

indicate low pathogenicity viruses found within highly pathogenic virus groups that were related to the Caspian Sea strains (presumably, highly pathogenic viruses appeared because of reassortment with low pathogenicity viruses). Red box indicates virus strains from Romania closely related to the Caspian Sea strains. Closed circles indicate the highly pathogenic avian influenza H5N1 virus strains isolated from dead Caspian terns on Maliy Zhemchuzhniy Island, Caspian Sea, Russia in May 2022. Closed triangles indicate Egyptian-like virus strains from Russia isolated in 2020. Sequences were obtained from the GISAID EpiFlu database (https://www.gisaid.org). Scale bar indicates nucleotide substitutions per site. HPAI, high pathogenicity avian influenza; LPAI, low pathogenicity avian influenza.

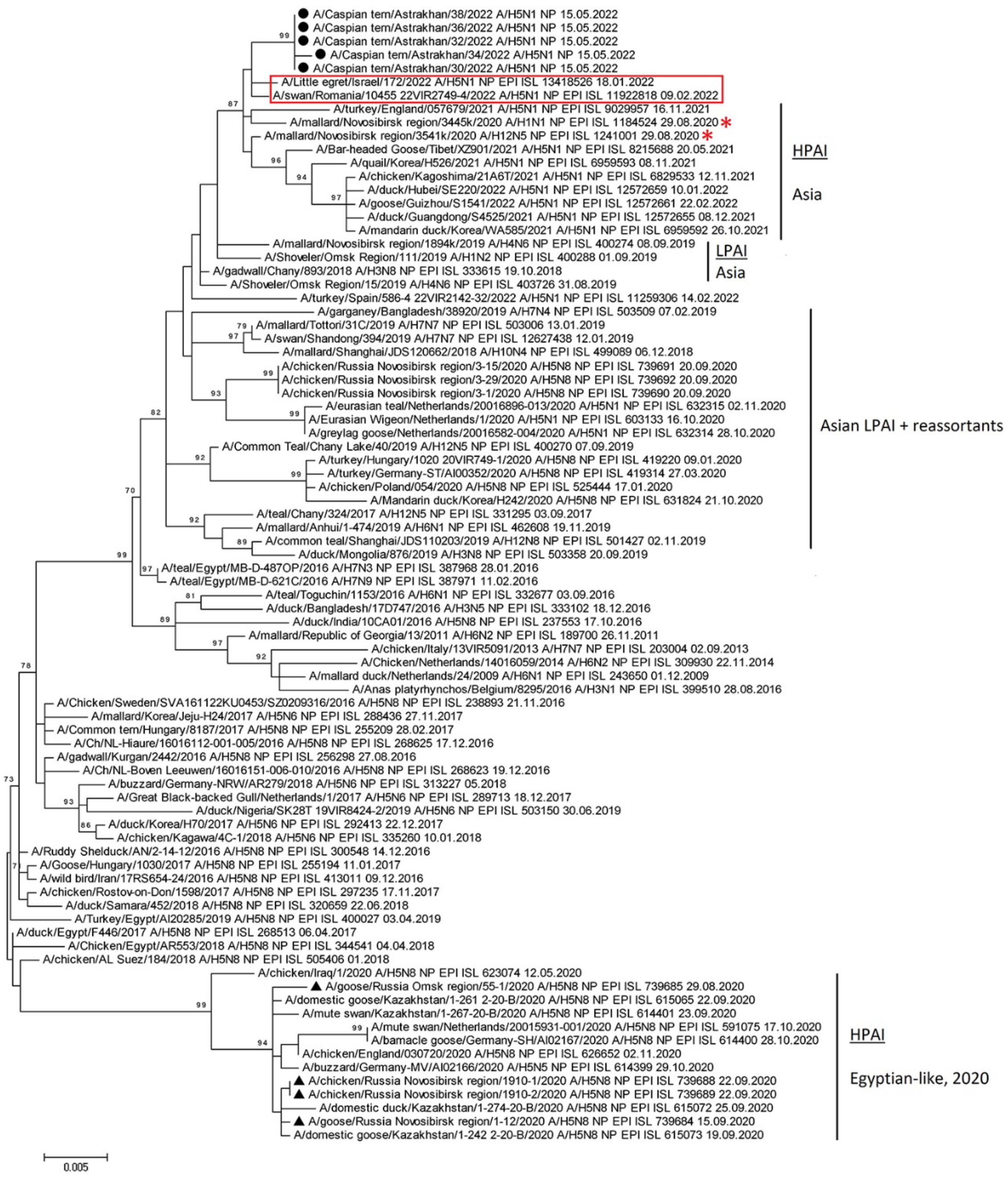

Appendix 1 Figure 7. Phylogenetic analysis of nucleoprotein gene segments of avian influenza viruses. Trees were constructed by using the maximum-likelihood method. Closed circles indicate the highly pathogenic avian influenza H5N1 virus strains isolated from dead Caspian terns on Maliy Zhemchuzhniy Island, Caspian Sea, Russia in May 2022. Red box indicates virus strains from Israel and Romania closely related to the Caspian Sea strains. Red asterisks indicate low pathogenicity viruses found within highly pathogenic virus groups that were related to the Caspian Sea strains (presumably, highly pathogenic viruses appeared because of reassortment with low pathogenicity viruses). Closed triangles

indicate Egyptian-like virus strains from Russia isolated in 2020. Sequences were obtained from the GISAID EpiFlu database (https://www.gisaid.org). Scale bar indicates nucleotide substitutions per site. HPAI, high pathogenicity avian influenza; LPAI, low pathogenicity avian influenza.

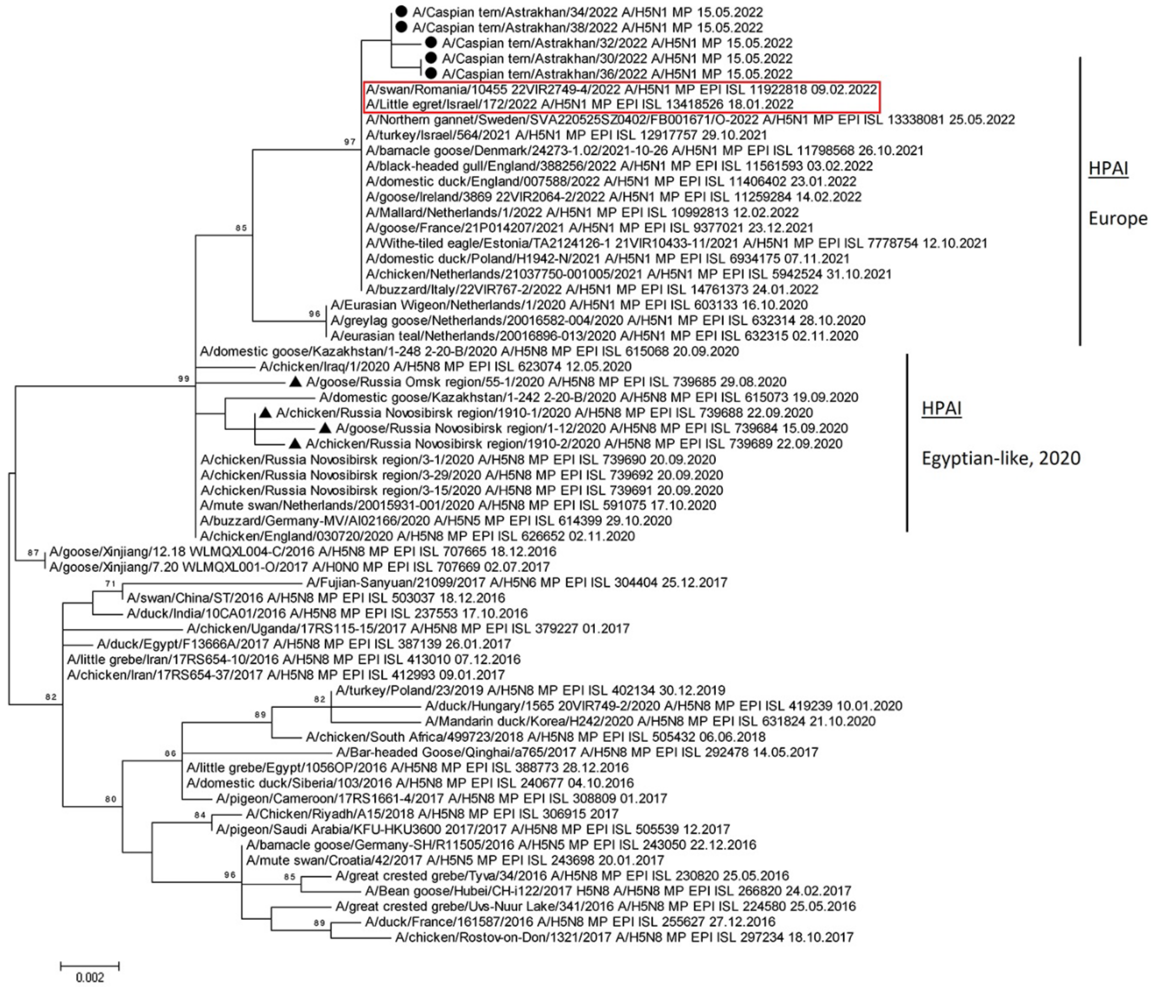

Appendix 1 Figure 8. Phylogenetic analysis of matrix protein gene segments of avian influenza viruses. Trees were constructed by using the maximum-likelihood method. Closed circles indicate the highly pathogenic avian influenza H5N1 virus strains isolated from dead Caspian terns on Maliy Zhemchuzhniy Island, Caspian Sea, Russia in May 2022. Red box indicates virus strains from Israel and Romania closely related to the Caspian Sea strains. Closed triangles indicate Egyptian-like virus strains from Russia isolated in 2020. Sequences were obtained from the GISAID EpiFlu database (https://www.gisaid.org). Scale bar indicates nucleotide substitutions per site. HPAI, high pathogenicity avian influenza; LPAI, low pathogenicity avian influenza.

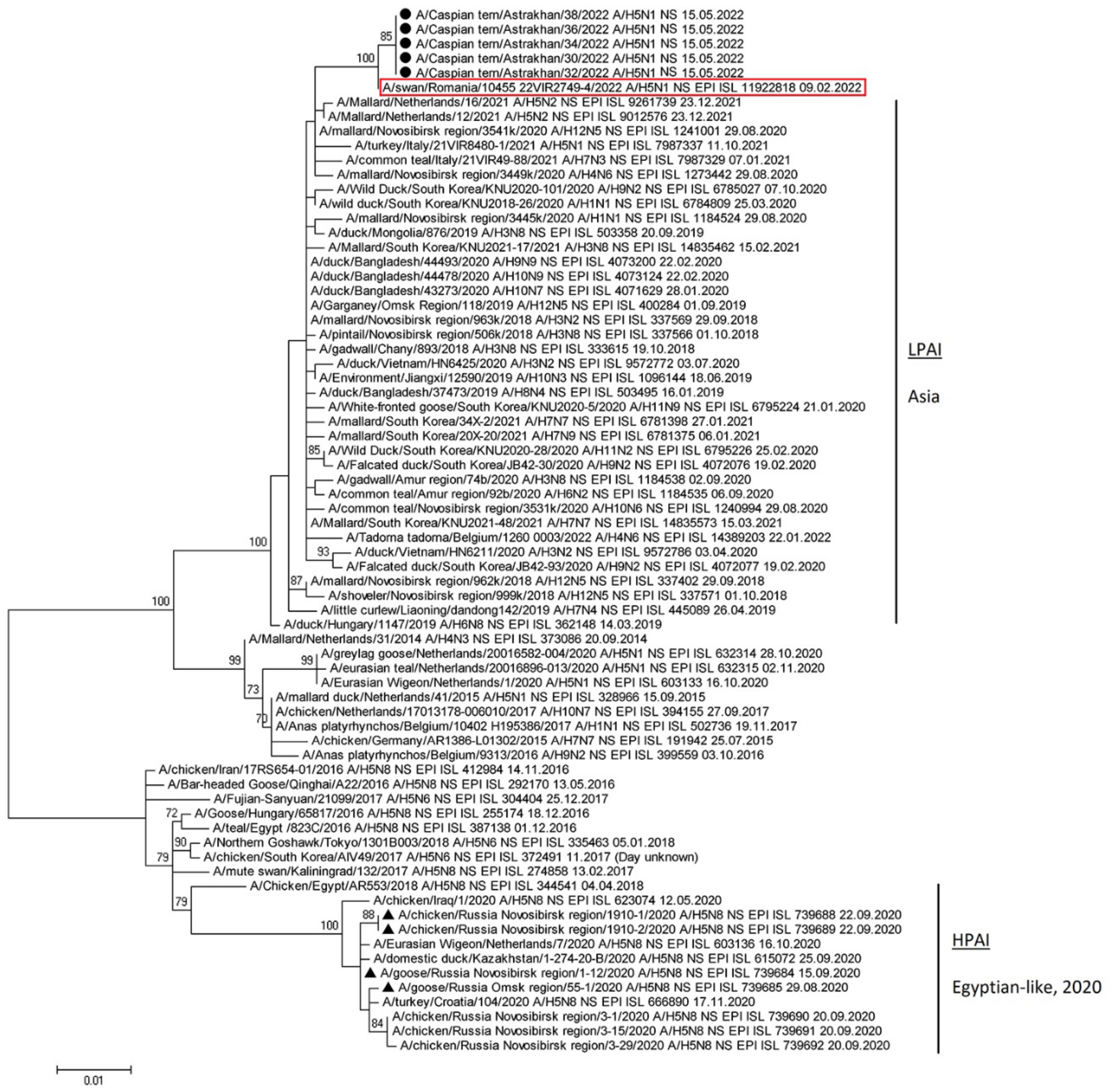

Appendix 1 Figure 9. Phylogenetic analysis of nonstructural protein gene segments of avian influenza viruses. Trees were constructed by using the maximum-likelihood method. Closed circles indicate the highly pathogenic avian influenza H5N1 virus strains isolated from dead Caspian terns on Maliy Zhemchuzhniy Island, Caspian Sea, Russia in May 2022. Red box indicates virus strains from Israel and Romania closely related to the Caspian Sea strains. Closed triangles indicate Egyptian-like virus strains from Russia isolated in 2020. Sequences were obtained from the GISAID EpiFlu database (https://www.gisaid.org). Scale bar indicates nucleotide substitutions per site. HPAI, high pathogenicity avian influenza; LPAI, low pathogenicity avian influenza.

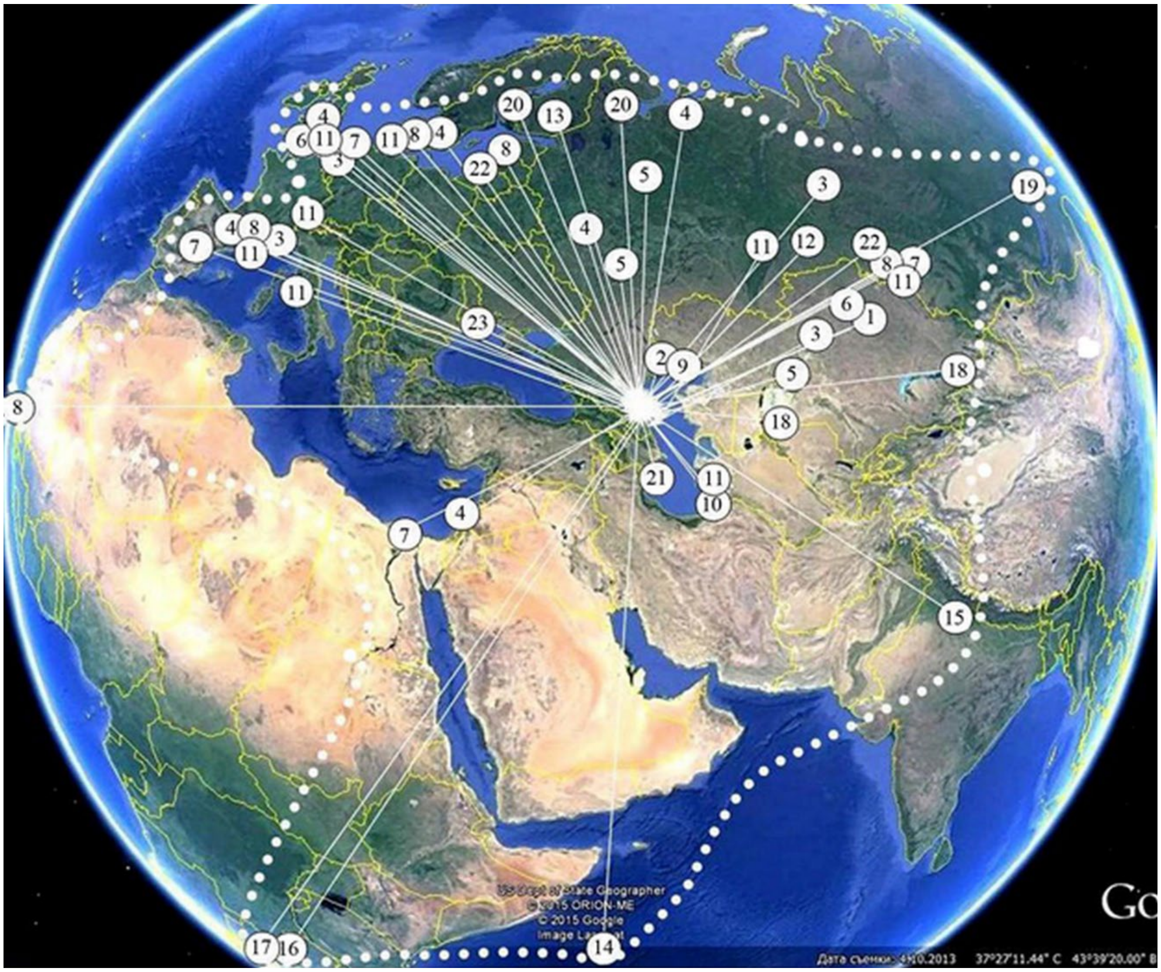

Appendix 1 Figure 10. Reconstructive analysis of flight routes according to data from the Russian Bird Ringing Center. Numbers represent different bird species: 1, *Anser anser*; 2, *Cygnus olor*; 3, *Anas platyrhynchos*; 4, *Anas crecca*; 5, *Anas strepera*; 6, *Anas penelope*; 7, *Anas acuta*; 8, *Anas querquedula*; 9, *Anas clypeata*; 10, *Netta rufina*; 11, *Aythya ferina*; 12, *Bucephala clangula*; 13, *Pandion haliaetus*; 14, *Arenaria interpres*; 15, *Himantopus himantopus*; 16, *Calidris ferruginea*; 17, *Calidris alba*; 18, *Larus ichthyaetus*; 19, *Larus minutus*; 20, *Larus fuscus*; 21, *Larus cachinnans*; 22, *Larus canus*; 23, *Larus genei*. Image and descriptions are from (*4*), licensed under CC BY 4.0 (https://creativecommons.org/licenses/by/4.0).
